# Humidified versus nonhumidified low-flow oxygen therapy in children with Pierre-Robin syndrome: A randomized controlled trial

**DOI:** 10.1097/MD.0000000000030329

**Published:** 2022-09-23

**Authors:** Xin Zhang, Aijuan Fan, Yingfei Liu, Li Wei

**Affiliations:** a Surgical Intensive Care Unit, Children’s Hospital of Nanjing Medical University, Nanjing City, Jiangsu Province, China.

**Keywords:** care, children, humidification, nursing, oxygen, Pierre-Robin syndrome

## Abstract

Humidification is an important process in clinical oxygen therapy. We aimed to evaluate the effects and safety of humidified versus nonhumidified low-flow oxygen therapy in children with Pierre-Robin syndrome.

**Methods:**

This study was an open-label, single-centered randomized controlled trial (RCT) with a parallel group design. The study protocol has been registered in Chinese Clinical Trial Registry (ChiCTR1900021584). The children were randomized to the humidified versus nonhumidified groups. Average arterial oxygen partial pressure (PaO_2_) and carbon dioxide partial pressure (PaCO_2_), incidence of ventilator-associated pneumonia (VAP), nasal cavity dryness, nasal mucosal bleeding and bacterial contamination of the humidified bottle, the cost of nasal oxygen therapy and duration of intensive care unit (ICU) stay were analyzed.

**Results:**

A total of 213 children with Pierre-Robin syndrome were included. There were no significant differences in the gender, age, weight, prematurity, duration of anesthesia and surgery duration of mandibular traction between humidified group and nonhumidified group (all *P* > .05). No significant differences in the average arterial PaO_2_ and PaCO_2_ level on the postoperative day 1, 2, and ICU discharge between humidified group and nonhumidified group were found (all *P* > .05). There were no significant differences in the incidence of nasal cavity dryness, nasal mucosal bleeding, bacterial contamination and VAP, the duration of ICU stay between humidified group and nonhumidified group (all *P* > .05). The cost of nasal oxygen therapy in the humidified group was significantly less than that of nonhumidified group (*P* = .013).

**Conclusions:**

Humidifying the oxygen with cold sterile water in the low-flow oxygen therapy in children may be not necessary. Future RCTs with lager sample size and rigorous design are warranted to further elucidate the effects and safety of humidified versus nonhumidified low-flow oxygen therapy.

## 1. Introduction

Oxygen therapy has been widely used for treatments in hospitals and home settings. Nasal cannula is the most commonly used oxygen inhalation device in clinical practice, and its utilization rate is as high as 70.52%.^[1]^ Humidification management is an important part of oxygen therapy care. As recommended by relevant oxygen therapy guidelines, there is currently no objective evidence to prove that humidification in 1~4 L/min oxygen therapy has significant clinical benefits, and there is a risk of infection in humidification.^[2–4]^ Most European and North American countries use nonhumidified oxygen for low-flow oxygen, while China and Japan still often perform humidified oxygen in clinical practice.^[5]^ The results of previous surveys^[6,7]^ have showed that 84.48% of clinical nurses in China will perform humidification in oxygen supply regardless of the oxygen flow rate. At present, there are still big differences in humidification management in clinical nursing practice amongst different countries. Understanding the effects and safety of humidification during oxygen therapy is of great significance to the prognosis of patients.

Pierre-Robin syndrome is a genetic disease with characteristics of facial deformities. The incidence of Pierre-Robin syndrome varies from 1/8500~1/14,000.^[8,9]^ Pierre-Robin syndrome mainly manifests as mandibular deformity, tongue that’s placed further back toward the throat, cleft palate, difficulty in breathing air, and limited humidification space for inhaled air.^[10]^ Children with Pierre-Robin syndrome generally may not tolerate milk intake and may experience breathing problems.^[11]^ Therefore, malnutrition, weight loss, and slow growth are common symptoms in children with Pierre-Robin syndrome. Clinically, mild Pierre-Robin syndrome can be treated conservatively, while moderate to severe Pierre-Robin syndrome generally requires mandibular traction to reduce airway obstruction, thereby improving pulmonary ventilation and feeding intolerance.^[12,13]^ In addition, it is worth noting that due to airway dysfunction, children need to further strengthen the oxygen support for before and after mandibular traction.^[14,15]^ At present, in clinical practice, after the child is weaned from the ventilator, a nasal cannula is usually used for additional oxygen support to meet the oxygen demand. Safe and effective oxygen support is very important for the prognosis of children with Pierre-Robin syndrome. Therefore, we conducted this randomized controlled trial (RCT) to compare the effectiveness and safety of the humidified versus nonhumidified low-flow oxygen therapy in children with Pierre-Robin syndrome, to provide reliable reference for more scientific and safe implementation of oxygen therapy care.

## 2. Methods

Our study has been registered in the Chinese Clinical Trial Registry (http://www.chictr.org.cn/) with registration number of ChiCTR1900021584. Besides, the study protocol had been published in the Journal of Clinical Nursing in the year of 2019 (PMID:31162860, DOI: 10.1111/jocn.14943).^[16]^ We aimed to perform and report this present RCT according to the statement of consolidated standards of reporting trials.^[17]^

### 2.1. Ethics

In this study, all related methods were performed in accordance with the relevant guidelines and regulations. Our study has been approved by the ethics committee of Children’s Hospital of Nanjing Medical University (201801163–1). Written informed consents had been obtained from all the parents or guardians of included children.

### 2.2. Patients

The children with Pierre–Robin syndrome treated in our hospital from May 1, 2019 to October 30, 2021 were recruited. The inclusion criteria in this present study were as following: children were diagnosed with Pierre-Robin syndrome; the age of children was <3 years old; the children underwent the surgery treatment of mandibular traction in our hospital; children received low-flow oxygen therapy (≤5 mL/min) with nasal cannula treatment during the stay at the intensive care unit (ICU); children did not have congenital diseases such as congenital heart disease; the guardians of children agreed to participate in this study. The exclusion criteria in this present study were as following: the guardians of children did not agree to participate in the study; children received high flow (>5 mL/min) heated and humidified oxygen therapy during the ICU stay.

### 2.3. Interventions

The included children were randomly distributed to the humidified group and nonhumidified group at a ratio of 1:1 by the method of allocation sequence table. The sequence of randomization was covered in opaque sealed envelopes and triggered when there were enrolled patients. We only set blinding design on the children to reduce the biases. All the humidified and nonhumidified interventions was performed in the surgical intensive care unit (SICU) of our hospital. All enrolled children underwent mandibular traction treatment with mechanical ventilation, and after being woke up from the anesthesia, the children would withdraw from the mechanical ventilation and underwent nasal oxygen therapy in the SCIU. For humidified group, the oxygen was routinely humidified with disposable bottle containing sterile water (Weigao, China). For nonhumidified group, the oxygen was not humidified with disposable bottle containing sterile water (Weigao, China). The nasal oxygen therapy continued when the children were discharged from ICU. We made the follow-up until the discharge of children with Pierre-Robin syndrome.

### 2.4. Outcome indicators

The blood gas was routinely analyzed daily during the stay of ICU. The doctor took 2 mL aerial blood for blood gas analysis in the test machine (HX/BG-800, Hengsheng, China) daily. Oxygen partial pressure (PaO_2_) and carbon dioxide partial pressure (PaCO_2_) were evaluated.

We assessed the potential complications associated with the oxygen therapy, including: incidence of ventilator-associated pneumonia (VAP): the diagnosis and treatment of VAP was based on the related clinical guidelines^[18,19]^; Incidence of nasal cavity dryness: The nasal cavity dryness was routinely evaluated every 1 h by the nurse in charge, if the facet of nasal cavity was not wet and it was covered with booger, it was rated as nasal cavity dryness; Incidence of nasal mucosal bleeding: the nasal mucosa was routinely evaluated every 1 h by the nurse in charge to check if the mucosa was dry and showed signs of bleeding; Incidence of bacterial contamination of the humidified bottle: The humidified bottle containing sterile water was sent to the laboratory for bacterial culture when the children were discharged from SICU, and if the bacterial culture of humidified bottle was positive, then it was defined as bacterial contamination. Furthermore, the cost of nasal oxygen therapy was collected and recorded when the children discharged from SICU, including the cost of oxygen, nasal oxygen cannula and disposable humidified bottle. The duration of ICU stay was analyzed.

### 2.5. Statistical analysis

SPSS software (version 21.0; SPSS Inc., Chicago, IL) was used for data analysis. The numerical variable data were expressed as mean ± standard deviation, and the categorical variable data were expressed as a percentage (%). Continuous and categorical variables between groups were compared using parametric independent-samples *t* tests and chi-square tests, respectively. *P* < .05 was considered as being statistically significant difference in this present study.

## 3. Results

### 3.1. Children inclusion

**Figure 1.**
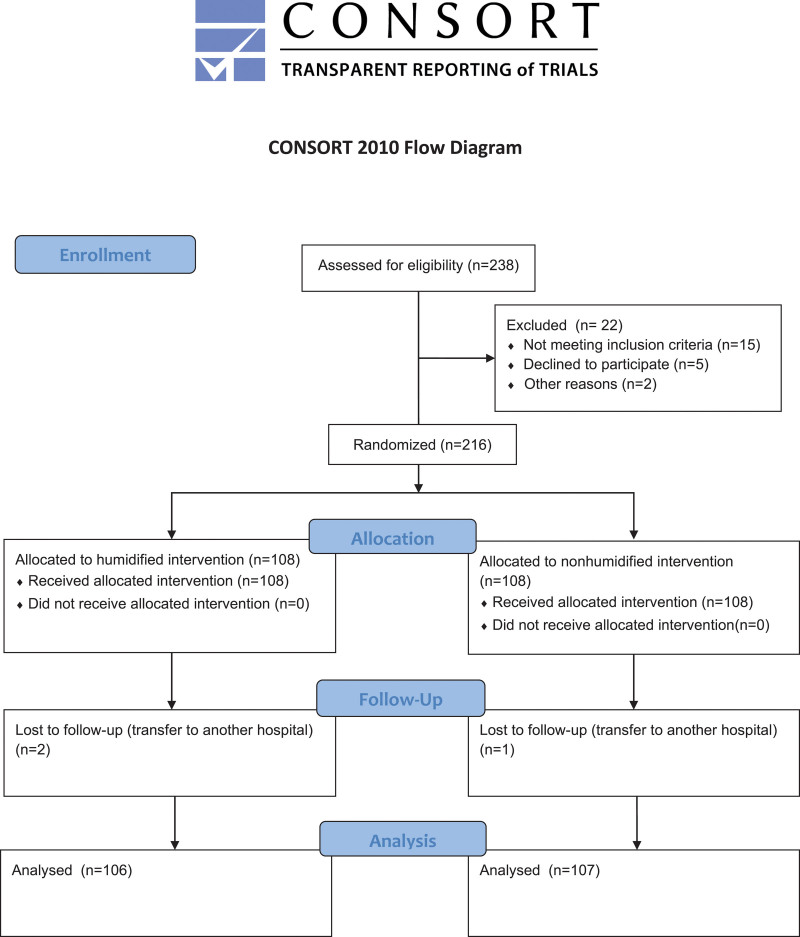
CONSORT flow diagram of patient inclusion. CONSORT = consolidated standards of reporting trials.

As indicated in Figure 1, a total of 238 children with Pierre-Robin syndrome were identified initially, 22 children were excluded at the initial screening, 216 children were randomized to the humidified group and nonhumidified group. 2 children in humidified group and 1 in nonhumidified group transferred to another hospital during the ICU stay. Finally, a total of 213 children with Pierre-Robin syndrome were included, of whom 106 children was allocated into the humidified group and 107 children into the nonhumidified group.

### 3.2. The characteristics of included children

As presented in Table 1, there were no significant differences regarding gender, age, weight, prematurity, duration of anesthesia and surgery duration of mandibular traction between humidified group and nonhumidified group (all *P* > .05), indicating that the baseline data of the 2 groups of children were comparable.

**Table 1:** The characteristics of included children with Pierre-Robin syndrome.

Items	Humidified group (n = 106)	Nonhumidified group (n = 107)	*t*/χ^2^	*P*
Male/female	61/45	59/48	1.022	.116
Age (mo)	3.44 ± 1.71	3.56 ± 1.04	1.168	.093
Weight (kg)	4.86 ± 2.05	4.77 ± 2.18	2.042	.069
Premature baby	18 (16.98%)	18 (16.82%)	1.955	.106
Duration of anesthesia (min)	120.17 ± 31.02	115.49 ± 40.11	21.495	.138
Surgery duration of mandibular traction (min)	98.18 ± 25.24	94.07 ± 26.44	16.208	.151

### 3.3. The changes of average arterial PaO_2_ and PaCO_2_

As shown in Table 2, there were no significant differences in the average arterial PaO_2_ and PaCO_2_ level on postoperative day 1, 2, and ICU discharge between humidified group and nonhumidified group (all *P* > .05).

**Table 2:** The changes of average arterial oxygen partial pressure (PaO_2_) and carbon dioxide partial pressure (PaCO_2_).

Items	Humidified group (n = 106)	Nonhumidified group (n = 107)	*t*	*P*
PaO_2_ on postoperative day 1 (mm Hg)	89.73 ± 9.95	90.11 ± 9.36	7.902	.145
PaO_2_ on postoperative day 2 (mm Hg)	94.09 ± 8.33	94.12 ± 7.15	6.398	.112
PaO_2_ on ICU discharge (mm Hg)	98.39 ± 4.11	98.25 ± 3.58	4.148	.177
PaCO_2_ on postoperative day 1 (mm Hg)	41.18 ± 7.18	41.02 ± 8.41	4.525	.095
PaCO_2_ on postoperative day 2 (mm Hg)	39.78 ± 7.01	40.13 ± 7.47	3.966	.183
PaCO_2_ on ICU discharge (mm Hg)	37.04 ± 5.93	37.54 ± 6.29	4.101	.096

ICU = intensive care unit.

### 3.4. The comparisons on the associated complications

As indicated in Table 3, there were no significant differences in the incidence of nasal cavity dryness, nasal mucosal bleeding, bacterial contamination and VAP between humidified group and nonhumidified group (all *P* > .05).

**Table 3:** The complications associated with the oxygen therapy.

Items	Humidified group (n = 106)	Nonhumidified group (n = 107)	*t*	*P*
Incidence of nasal cavity dryness	21 (18.87%)	20 (18.69%)	1.939	.108
Incidence of nasal mucosal bleeding	3 (2.83%)	4 (3.74%)	1.225	.061
Incidence of bacterial contamination of the humidified bottle	1 (0.94%)	2 (1.87%)	1.056	.095
Incidence of ventilator-associated pneumonia	1 (0.94%)	1 (0.93%)	1.048	.981

### 3.5. The cost of nasal oxygen therapy and duration of ICU stay

As showed in Table 4, the cost of nasal oxygen therapy in the humidified group was significantly more than that of nonhumidified group (*P* = .013), there was no significant difference in the duration of ICU stay between humidified group and nonhumidified group (*P* = .098).

**Table 4:** The cost of nasal oxygen therapy and duration of ICU stay.

Items	Humidified group (n = 106)	Nonhumidified group (n = 107)	*t*	*P*
Cost of nasal oxygen therapy (RMB)	411.85 ± 135.23	281.36 ± 85.02	47.227	.013
Duration of ICU stay (d)	4.29 ± 2.88	4.12 ± 3.17	3.405	.098

ICU = intensive care unit.

## 4. Discussion

At present, sterile water is routinely used for humidification during low-flow oxygen inhalation of nasal cannula in the clinical nursing practice in China.^[20]^ Besides, Chinese nursing education textbooks still recommend the use of sterile water for low-flow oxygen humidification.^[21]^ However, several recent studies^[22–24]^ have concluded that there is no need for regular humidification of low flow oxygen. It must be noted that most of the relevant studies are conducted in the adult population, and there are few studies in children. Therefore, we believe that more studies are necessary to investigate the role of humidified low-flow oxygen in the pediatric population to provide more insights into the clinical practice. The results of this study showed that there was no significant difference in the effect of oxygen therapy between humidified and nonhumidified medium and low flow nasal cannula oxygen, and no significant difference in the incidence of VAP, nasal cavity dryness, nasal mucosal bleeding, bacterial contamination of the humidified bottle and the duration of ICU stay between humidified group and nonhumidified group, yet the cost of nonhumidified nasal oxygen therapy is significant less than that of humidified group. Therefore, the nonhumidified oxygen therapy may be more appropriate for clinical practice with consideration to equal effects and less cost.

Previous studies^[25,26]^ have found that when oxygen is inhaled, humidification of oxygen can prevent dry oxygen from irritating the respiratory mucosa, avoid the incidence of nasal dryness and bleeding, and reduce the discomfort of oxygen inhalation. However, some studies^[27,28]^ have pointed out that there are no significant differences in improving the comfort of oxygen inhalation of patients between the humidified and nonhumidified oxygen inhalation, and there is no solid evidence to indicate that to perform moisturized oxygen inhalation on patients who need to inhale low-to-medium flow oxygen. Under normal circumstances, the upper respiratory tract mucosa of the human body can warm, humidify, filter, and clean the inhaled air.^[29,30]^ Oxygen is not easily soluble in water at room temperature, and ordinary cold water has a limited humidification effect on oxygen. Previous studies^[31,32]^ have pointed out that the decrease in oxygen humidity can be compensated by increasing the humidity in the hospital room. Therefore, the routine humidification by cold sterile water may be not effective and necessary for low flow oxygen therapy.

During normal breathing process, the respiratory tract itself has a certain ability to adjust the humidity and temperature of inhaled oxygen. It can heat and humidify the gas, and the inhaled gas can reach 37°C and the relative humidity of 100% at the carina, which meets the physiological needs of the human body.^[33]^ Previous research results^[34,35]^ have shown that medical oxygen inhaled through nasal cannula only accounts for 2.4% to 19% of the moisture. The lack of humidification produced by nonhumidified medical oxygen can be compensated by adjusting the ambient air humidity. The respiratory tract has the function of humidifying and regulating the inhaled medium and low flow oxygen, while the humidified medium and low flow oxygen has no obvious effect on preventing the deterioration of nasal mucociliary function, mucus properties and pulmonary function.^[36–38]^ Still, future theoretical and clinical studies are needed to evaluate the feasibility of nonhumidified low-to-medium flow oxygen therapy.

The results of previous studies^[39,40]^ suggest that oxygen therapy with nonhumidified low-flow nasal cannula has no influence on the comfort of oxygen inhalation and the effect of oxygen therapy, which is consistent with the results of this study. There is no obvious correlation between the comfort of patients and whether the oxygen is humidified.^[41]^ When inhaling oxygen with a medium and low flow nasal cannula, the tidal volume exceeds 16% the volume of oxygen inhaled, and the warming and humidification of the nasal mucosa can fully humidify the inhaled air to meet physiological needs.^[42]^ The traditional oxygen humidifying bottle uses humidifying liquid to inhale oxygen after being humidified.^[43]^ The bacteria grown in the humidifying liquid collide with the humidifying liquid through a large number of oxygen bubbles, forming aerosols that are sucked into the alveoli and cause infection.^[44]^ The results of previous study^[45]^ have shown that the positive rate of bacterial contamination in the humidification bottle can be as high as 9.15% after the patient’s low-flow oxygen inhalation for 24 h. The low and medium flow oxygen supply in non-humidification contributes to respiratory infections caused by bacterial contamination of the humidification bottle.^[6]^ The results of this study did not show a significant difference in pulmonary infection between children with humidified and nonhumidified oxygen, which may be related to the small sample size of the study.

Several limitations in this present study must be considered. Firstly, considering the nature of intervention and limited by human resource, it is difficult to blind the personnel during the intervention and outcome assessment, therefore biases may exist in the outcome assessment. Secondly, our study was conducted in one single SICU, the sample size was small, and our study might be underpowered to detect the group differences of some variables. Therefore, future RCTs with lager sample size, rigorous design in different areas and populations are needed to further elucidate the effects and safety of humidified versus nonhumidified low-flow oxygen therapy in clinical practice.

## 5. Conclusions

In conclusions, the results of this RCT showed that humidified versus nonhumidified low-flow oxygen therapy did not show positive effects in children with Pierre–Robin syndrome, and nonhumidified low-flow oxygen therapy had less medical cost, which may be more appropriate for clinical nursing care in oxygen practice in children. In the future, the role of humidified versus nonhumidified low-flow oxygen therapy needs to be investigated by more rigorous and high-quality studies, to provide useful basis for the clinical humidified practice of oxygen delivery.

## Author contributions

**Conceptualization:** Xin Zhang.

**Data curation:** Xin Zhang, Aijuan Fan, Li Wei.

**Formal analysis:** Aijuan Fan, Yingfei Liu, Li Wei.

**Investigation:** Aijuan Fan, Yingfei Liu, Li Wei.

**Resources:** Yingfei Liu.

**Project administration:** Xin Zhang, Aijuan Fan.

**Software:** Xin Zhang, Li Wei.

**Supervision:** Xin Zhang, Yingfei Liu.

**Validation:** Xin Zhang, Aijuan Fan.

**Visualization:** Xin Zhang, Li Wei.

**Writing – original draft:** Aijuan Fan, Yingfei Liu, Li Wei.

**Writing – review & editing:** Li Wei.
